# Association of Anaemia With Higher Mortality and Disability After Subarachnoid Haemorrhage: A Single-Centre Cohort Study

**DOI:** 10.7759/cureus.83031

**Published:** 2025-04-26

**Authors:** Priyavarshini Ramesh, Mark Horner, Marcus Abbawy, Finlay Holden, Suresh Renukappa, Subashini Suresh, Cyril Chacko, Randeep Mullhi, Sree Chaitanua Rudrapatma Sathyamurthy, Tonny Veenith

**Affiliations:** 1 Department of Critical Care Medicine, Guy's and St Thomas' NHS Foundation Trust, London, GBR; 2 Department of Anaesthesia and Critical Care, University Hospitals Birmingham NHS Foundation Trust, Birmingham, GBR; 3 Department of Anaesthesia, University Hospitals Birmingham NHS Foundation Trust, Birmingham, GBR; 4 Department of Critical Care and Acute Care Medicine, The Royal Wolverhampton NHS Trust, Wolverhampton, GBR; 5 Faculty of Science and Engineering, University of Wolverhampton, Wolverhampton, GBR; 6 Department of Critical Care Medicine, Dr B.R. Ambedkar Medical College and Hospital, Bengaluru, IND; 7 Acute Care Institute, Research Institute of Healthcare Sciences, University of Wolverhampton, Wolverhampton, GBR

**Keywords:** acute subarachnoid haemorrhage, anaemia, blood transfusion, intracerebral aneurysms, neurointensive care unit

## Abstract

Background: Critical care management in subarachnoid haemorrhage (SAH) aims to facilitate neuroprotection and prevent secondary neurological insults. Anaemia after SAH leads to reduced cerebral oxygen delivery and poor outcomes. This study aimed to investigate the association between anaemia and mortality and morbidity outcomes in patients with SAH in tertiary centres, as well as evaluate the impact of blood transfusions on outcomes in anaemic SAH patients.

Methods: We performed a retrospective study of 987 patients with SAH at a tertiary neurocritical care centre between September 2016 and September 2018. Data were collected on baseline characteristics, World Federation of Neurosurgical Societies SAH grade, secondary insults such as anaemia and hydrocephalus, and units of blood transfused. The primary outcome was to investigate the correlation between anaemia and 28-day Modified Rankin Scale (MRS) score. Secondary outcomes were delayed neurological deficits, length of stay and death in the intensive care unit (ICU). Anaemia was defined as haemoglobin (Hb) ≤ 95 g/L, and severe anaemia as Hb≤80 g/L.

Results: Of the patients, 28.7% had anaemia and 12% had severe anaemia. Anaemia after SAH was associated with an increased risk of death or severe disability (p<0.001), worse survival outcomes (p<0.001) and increased length of stay in ICU (p<0.001).

Conclusions: Anaemia after SAH is associated with a significant increase in mortality and morbidity and should be monitored closely and corrected.

## Introduction

Subarachnoid haemorrhage (SAH) is associated with high mortality and morbidity and has a profound impact on individuals, families, and society [1,2]. It often results in admission to intensive care for neuroprotection, prevention of secondary insults, and delayed cerebral ischaemia [3]. Secondary insults after an SAH affect long-term cognitive and neurological recovery [3,4]. Determinants of poor outcome after SAH include age, the aneurysmal aetiology (trauma, AV malformation, cryptogenic bleeding, aneurysm size), free haemoglobin load, consciousness at presentation, and complications afterwards [5]. Neurological disability after the ictus is propagated by further insults to the brain due to critical illness, worsening outcomes in the entire course of the disease [6].

Studies have suggested anaemia (haemoglobin (Hb) <10 g/L) occurs in up to 30% of SAH patients and leads to poorer outcomes [7-10]. Brain injury models suggest that in SAH, there is diffusion cerebral hypoxia in the absence of classic hypoxia, meaning regions within the brain still lack oxygen despite oxygenation levels appearing normal overall, with endothelial dysfunction and perivascular oedema [11]. This supports the belief that low Hb after SAH leads to reduced cerebral oxygen delivery and, consequently, poorer clinical outcomes. Hence, the pathophysiology of brain injuries and delayed cerebral ischaemia leads to an increased sensitivity to lower oxygen delivery, and timely blood transfusions may reduce the associated mortality and morbidity [6]. Thus, optimising oxygen delivery to at-risk brain regions could improve neurocognitive outcomes after SAH.

Physiological responses to anaemia include a short-term decrease in blood viscosity and the release of nitric oxide and hypoxia-inducible factor (HIF), which stimulate vasodilation, partially compensating for the harmful effects of acute anaemia. Studies in healthy individuals have shown that such compensatory mechanisms can maintain adequate cerebral oxygen delivery in anaemia as low as Hb of 50 g/L [12]. They also show that the symptoms of cerebral hypoxia, such as fatigue and amnesia, can be reversed quickly by correcting anaemia with an autologous transfusion. However, studies in the critically ill have indicated that blood transfusions such as these can be associated with negative complications such as liver dysfunction and increased rates of mortality [13,14].

Until the recent SaHaRa trial [15], good-quality data on transfusion thresholds were sparse, specifically after a SAH. This study aims to investigate the adverse outcomes associated with anaemia and the optimal haemoglobin threshold for triggering transfusions in SAH patients. Abnormal cerebral vasoreactivity and hyper- or hypotension with tissue hypoxia tend to interfere with anaemia-induced vasodilation as a compensatory mechanism, complicating SAH management [6].

Diffuse microvascular dysfunction after SAH, where impaired blood flow occurs in the microcirculation leading to ischaemia, requires a higher perfusion pressure and good oxygen delivery to prevent long-standing cerebral damage [11]. At-risk regions are poorly perfused areas of the brain at risk of developing infarcts when challenged by anaemia, potentially resulting in irreversible brain damage. All of these mechanisms can alter the balance towards secondary cerebral insult in a patient with SAH.

National guidelines NG24 and NG228 [16,17] recommend a target Hb range of 80-100 g/L in patients with SAH, but this is a wide range, and there is significant ambiguity as to whether patients with SAH should be given red blood cell transfusion (RBCT) at the upper-limit, more liberal threshold of 100g/L or the lower-limit, more restrictive threshold of 80g/L. There is a paucity of good-quality evidence on anaemia as a variable that predicts death or disability for people with SAH [17], which might go on to provide a basis for recommendations of RBCT thresholds in such patients.

Our study aimed to examine the timing of anaemia and the additional impact it causes. Anaemia was defined in the study as Hb ≤ 95 g/L for two reasons. Firstly, it was lower than the defined liberal threshold for transfusion of the <100 g/L SAHaRA trial, and secondly, the patient admitted to critical care is often anaemic due to intravenous fluids, multiple venesections, and acute on chronic anaemia of critical illness [18-20]. In the SAHaRA trial, over the first 21 days of hospitalisation, a liberal and restrictive transfusion did not show a reduced risk of an unfavourable neurological outcome at 12 months [15].

This study was presented as an e-poster at the Intensive Care Society’s State of the Art Conference 2021, 6-8 December 2021.

## Materials and methods

This was a longitudinal retrospective single-centre cohort study at a tertiary neurocritical care centre, the Queen Elizabeth Hospital, Birmingham, West Midlands, United Kingdom. Between September 2016 and September 2018, 987 patients with SAH were admitted directly or referred via a local district hospital. Data were collected on these patients from electronic medical records and relevant imaging in the form of computed tomography (CT) scans, all reported on by a consultant radiologist. This time period was useful to assess pre-COVID management, where the intensive care unit (ICU) was less pressured in resource allocation.

The Strengthening the Reporting of Observational studies in Epidemiology (STROBE) guidelines were used in preparation for this study. The study used routinely collected data; no intervention was performed, and patients were not contacted outside their routine clinical care. Therefore, specific ethical approval was not required, and patient consent was waived in line with guidance from the UK Health Research Authority and the UK Policy Framework for Health and Social Care Research (https://hra-decisiontools.org.uk/ethics/EngresultN1.html).

Inclusion and exclusion criteria

Inclusion criteria comprised all patients aged over 18 years with a confirmed diagnosis of SAH. Exclusion criteria included conditions that might confound the need for red blood cell transfusion (RBCT), such as haematological malignancies. Haematological malignancies, such as acute lymphoblastic leukaemia were excluded because they would trigger a patient-specific transfusion threshold that would change blood management in the SAH patient for that unique patient and not be generalisable to and reproducible for a larger cohort of patients with SAH. Our study aimed to investigate whether anaemia is associated with poorer neurological outcomes and increased morbidity and mortality in patients with SAH.

Data collection

Data were collected on baseline characteristics, the severity of the SAH, as per the World Federation of Neurosurgical Societies grading system, secondary insults such as the occurrence of hypoxia, hypotension, anaemia, and hydrocephalus, and units of blood transfused (if any). Hypotension, most commonly due to Cushing's variant, was classified as a secondary outcome due to the associated risk of cardiovascular collapse. The primary outcome was a 28-day Modified Rankin Scale (MRS) score (Table 1). Secondary outcomes were delayed neurological deficits, length of stay, and death in the ICU.

**Table 1:** Modified Rankin Score (at 28 days)

Score	Classification
0	No symptoms
1	No significant disability despite symptoms
2	Slight disability but able to carry out ADLs
3	Moderate disability but able to walk independently
4	Moderately severe disability; unable to attend to bodily needs independently
5	Severe disability; bed-bound
6	Death

Definitions

Anaemia was defined in the study as Hb ≤ 95 g/L. Hypoxia was defined as instances of oxygen saturation (SpO2) < 92% and hypotension as systolic blood pressure (SBP) < 80 mmHg, and also collected data for counts of SBP being 80-110 mmHg. Hydrocephalus was defined based on the screening of radiological reports. 

Data analysis

All analyses were performed with IBM SPSS Statistics for Windows, Version 22.0 (Released 2013; IBM Corp., Armonk, New York, United States). All variables were considered in univariate analysis, and p<0.05 was defined as statistically significant. Data are presented as mean (standard deviation) for continuous variables and percentages (numbers) for categorical variables. The relative risk of outcomes was analysed with linear regression according to the occurrence, severity, and timing of anaemia, hypoxia, and hypotension.

## Results

**Figure 1:**
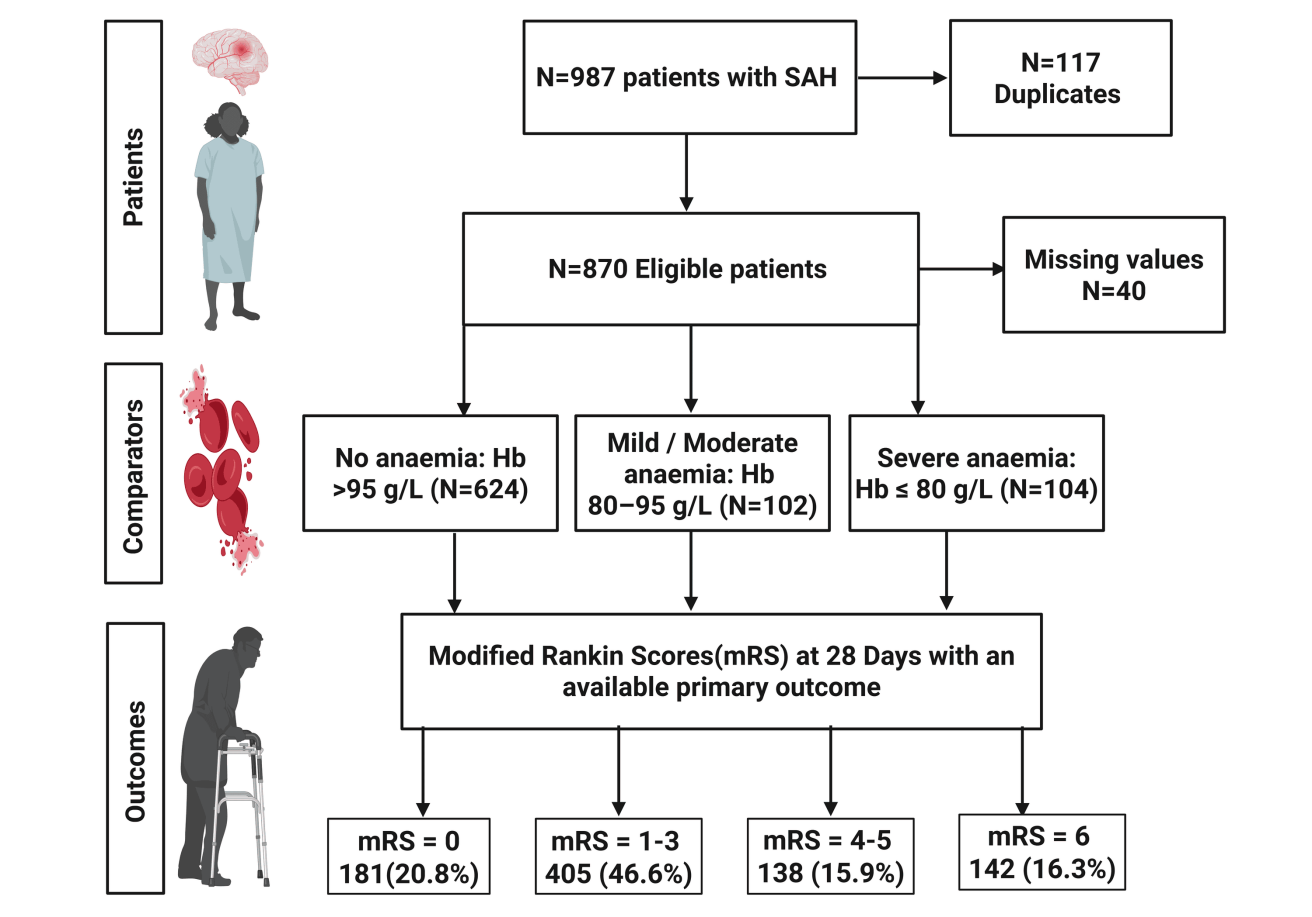
CONSORT diagram CONSORT: Consolidated Standards of Reporting Trails

A total of 987 patients with SAH were considered based on electronic neurosurgical referrals made to the hospital, out of which 870 were eligible for this study after removing duplicates and applying exclusion criteria, as demonstrated in Figure 1. Of these 870, 40 were excluded due to missing data, and hence, the final cohort was 830.

Female patients accounted for 519 (59.7%) of the study population. The mean age on admission was 57 (SD 14.4) years. The median length of stay in the ICU was 4.13 days (IQR 0.00-8.90, mean 8.42, SD 15.1). A total of 795 (91.4%) patients survived to discharge from the ICU, of which 497 (57.1%) survived to discharge from the hospital to home. Of those who survived discharge from hospital to home, 432 (69.7%) patients did not have anaemia during admission. The baseline variables and outcomes are provided in Table 2. 

**Table 2:** Baseline characteristics of the study population according to the severity of anaemia (N=830) WFNS: World Federation of Neurological Surgeons

Variables	No anaemia: Hb >95 g/L (n=624)	Mild/moderate anaemia: Hb 80–95 g/L (n=102)	Severe anaemia: Hb ≤ 80 g/L (n=104)
Sex, n (%)			
Female	364 (58.3%)	68.0 (66.7%)	65.0 (62.5%)
Male	260 (41.7%)	34.0 (33.3%)	39.0 (37.5%)
Age on Admission			
Mean (SD)	57.0 (13.5)	56.7 (13.7)	55.8 (16.0)
Median (IQR)	57.0 (47.35-66.65)	56.0 (46.1-65.9)	55.0 (43.0-67.0)
(min, max)	(18.0, 94.0)	(30.0, 84.0)	(17.0, 85.0)
Counts of systolic BP 80-110			
Mean (SD)	8.82 (15.2)	19.3 (28.1)	36.5 (54.7)
Median (IQR)	3.0 (0 – 8)	8.0 (0-19.75)	10.5 (0-38.4)
Range (min, max)	(0,148)	(0,161)	(0,276)
Counts of systolic blood pressure < 80			
Mean (SD)	0.0321 (0.231)	0.176 (0.916)	0.567 (1.80)
Median (IQR)	0 (0-0)	0 (0-0)	0 (0-0)
Range (min, max)	(0,3.00)	(0,8.00)	(0,15.0)
Counts of hypoxaemia (SpO2 < 92%)			
Mean (SD)	0.91 (4.60)	4.88 (15.3)	15.1 (46.2)
Median (IQR)	0 (0-0.25)	1.0 (0-3.0)	2.0 (0- 7.0)
Range (min, max)	(0,101)	(0,111)	(0,412)
Transfusion status			
No	624 (100%)	98.0 (96.1%)	44.0 (42.3%)
Yes	0 (0%)	4.0 (3.9%)	60.0 (57.7%)
WFNS, n (%)			
Grade 1	402 (64.4%)	31.0 (30.4%)	20.0 (19.2%)
Grade 2	73.0 (11.7%)	13.0 (12.7%)	17.0 (16.3%)
Grade 3	41.0 (6.6%)	4.0 (3.9%)	15.0 (14.4%)
Grade 4	51.0 (8.2%)	34.0 (33.3%)	23.0 (22.1%)
Grade 5	55.0 (8.8%)	19.0 (18.6%)	22.0 (21.2%)
Missing	2.0 (0.3%)	1.0 (1.0%)	7.0 (6.7%)
Death in ICU, n (%)			
No	583 (93.4%)	92 (90.2%)	82 (78.8%)
Yes	41 (6.6%)	10 (9.8%)	22 (21.2%)

A total of 250 (28.7%) patients admitted with SAH had anaemia during their admission, with 104 (12.0%) patients experiencing severe anaemia; 183 (21.0%) patients experienced anaemia within seven days of admission, of which 87 (10.0%) experienced severe anaemia. A total of 220 (25.3%) patients had a poor-grade SAH (WFNS 4 or 5) on admission, and 343 (39.4%) patients had secondary hydrocephalus. A total of 64 (25.6%) patients who experienced anaemia received RBCT during admission.

Primary outcome: 28-day MRS score

**Figure 2:**
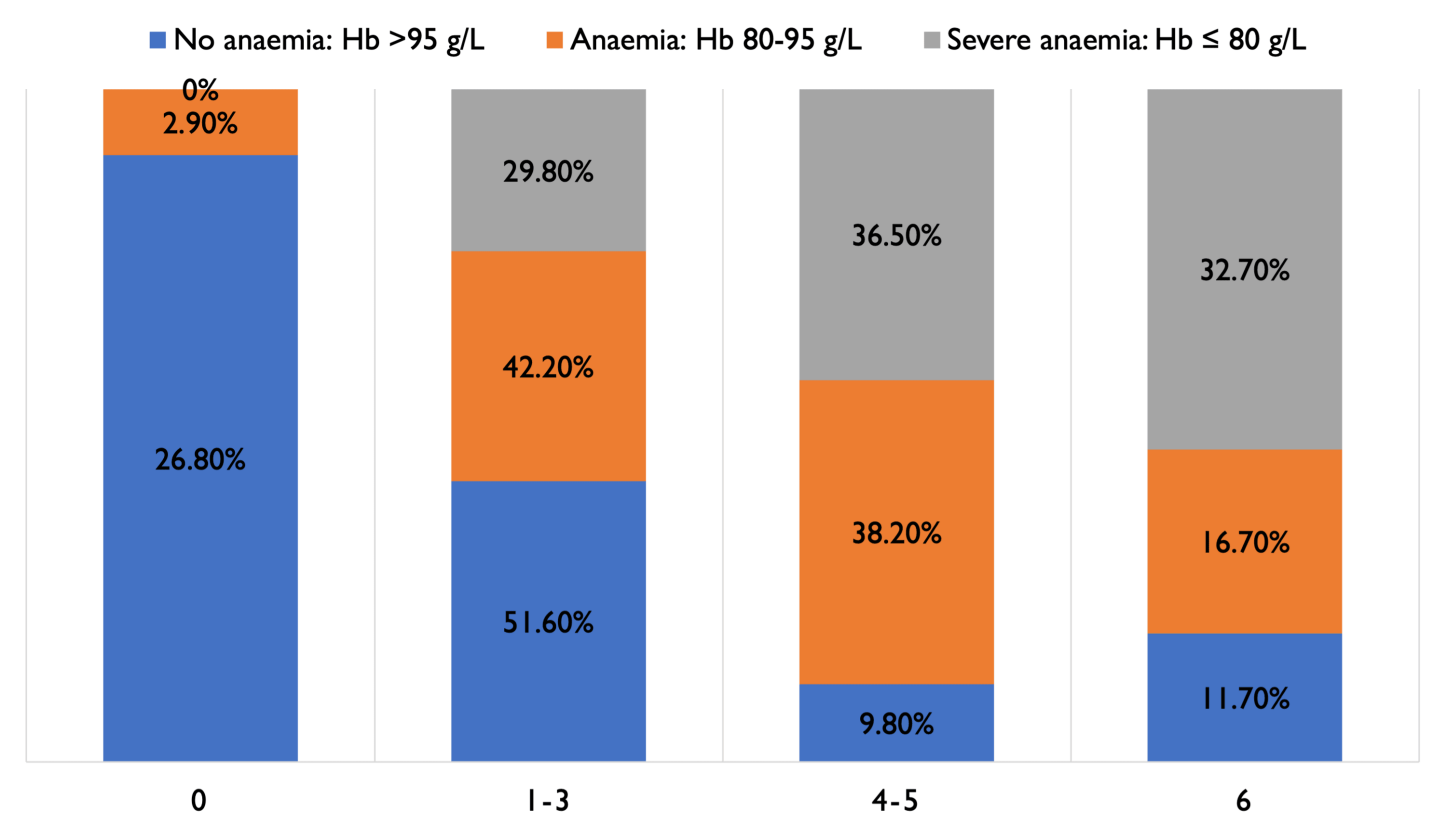
Comparison of the severity of anaemia and Modified Rankin Score

**Figure 3:**
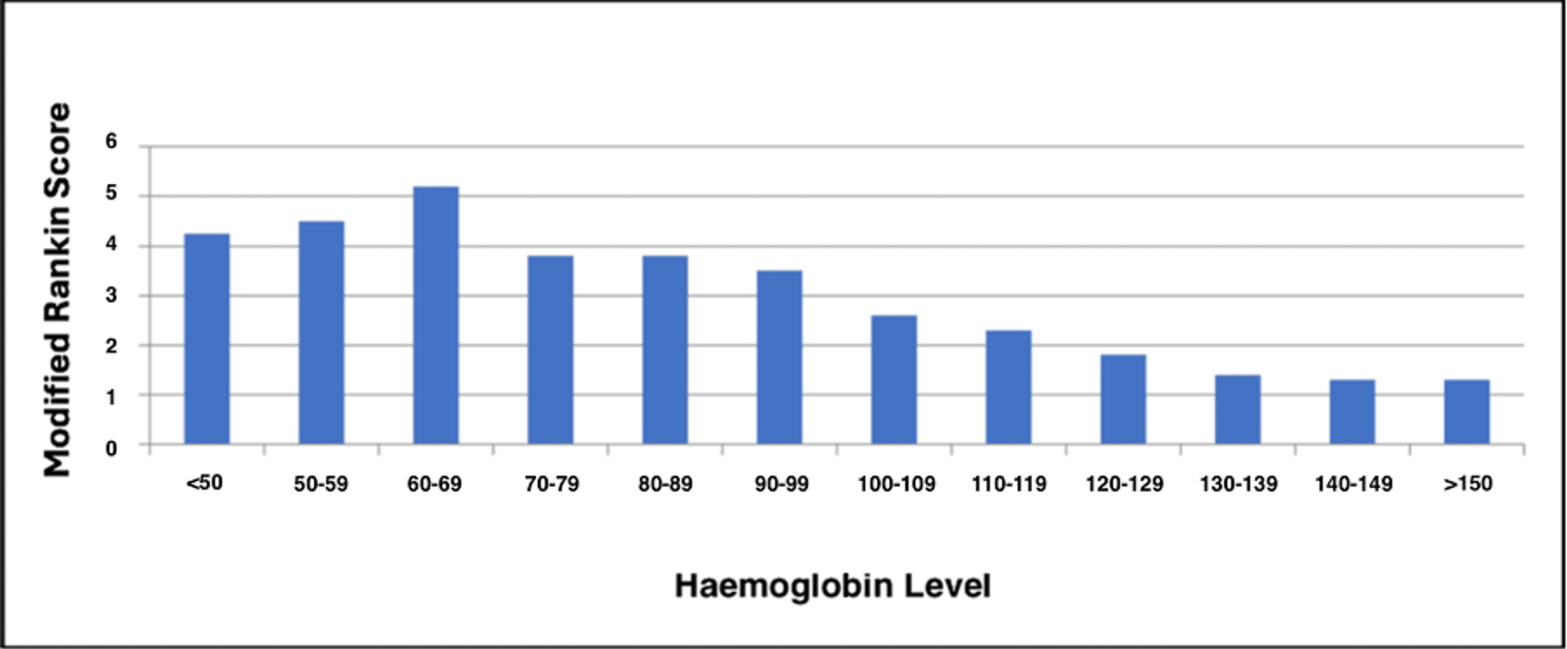
Haemoglobin levels against Modified Rankin Score

As shown in Figures 2-3, 61 (9.8%) patients with no anaemia had MRS scores of 4-5, indicating severe morbidity, compared to 39 (38.2%) in patients with moderate anaemia and 38 (36.5%) in patients with severe anaemia. Patients who had anaemia had a high risk of poor MRS outcome (4-6), indicating significant disability and/or death (Fisher's exact test, p<0.001). A total of 50 (49%) patients with moderate anaemia and 46 (44.2%) with severe anaemia had to be discharged to another hospital or neuro-rehabilitation centre, compared to 115 (18.4%) non-anaemic patients. 

Secondary outcomes

Adjusted for age and sex, severe anaemia was correlated with an increased risk of death in the ICU (relative risk (RR) 3.89, 95% confidence interval (CI) 2.17-6.83). The relative risk of death in the ICU was associated with severe anaemia post SAH, even after adjustment for severity of bleed and WFNS grade (RR 1.91, 95%CI 0.97-3.64). Univariate survival analysis of the lowest Hb, hypotension with a SBP of 80-100 mmHg, admission WFNS score, and occurrence of hydrocephalus were all statistically significant (Tables 3, 4). The summary of the results is outlined in Table 5.

**Table 3:** Univariate survival analysis * P values < 0.05 are significant

Variable	Coefficient	95% CI	P-value
Gender (Male/Female(reference))	0.9519	0.5980,1.5151	0.835
Age (years)	0.0106	-0.0058,0.0270	0.205
Lowest Hb	-0.0116	-0.0215,-0.0017	0.022 *
Number of days Hb <95g/L	-0.0035	-0.0262,0.0192	0.764
Counts of systolic BP 80-110 mmHg	-0.0585	-0.0877,-0.0292	<0.001 *
Counts of systolic BP < 80 mmHg	-0.0776	-0.3820,0.2269	0.618
Counts of hypoxia SpO2 < 92%	0.0038	-0.0017,0.0092	0.178
Did Hb fall < 95 (No/Yes(reference))	-0.3426	-0.8171,0.1319	0.157
WFN (1,2)=Good (reference) , (3,4,5)=Poor	1.5236	1.0091,2.0380	<0.001 *
Occurrence of hydrocephalus (Yes, No(reference))	-1.3110	-1.8646,-0.7573	<0.001 *
Modified Rankin Score 0,1,2 = Good : ≥3 =Poor	37.3958	-3.14e7,3.14e7	1.000

**Table 4:** Survival Analysis (Hazards Ratio) * P values < 0.05 are significant

Variable	Coefficient (Hazard Ratios)	95% CI	P-value
Gender (Male/Female(reference))	0.9519	0.5980-1.5151	0.835
Age (years)	1.0106	0.9942-1.0273	0.205
Lowest Hb	0.9885	0.9787-0.9983	0.022 *
Number of days Hb <95g/L	0.9965	0.9741-1.0194	0.764
Counts of systolic BP 80-110mmHg	0.9432	0.9160-0.9712	<0.001 *
Counts of systolic BP < 80	0.9254	0.6825-1.2547	0.618
Counts of hypoxia SpO2 < 92%	1.0038	0.9983-1.0093	0.178
Did Hb fall <95g/L (No/Yes(reference))	0.7099	0.4417-1.1410	0.157
WFN (1,2) =Good (reference), (3,4,5)=Poor	4.5885	2.7432-7.6752	<0.001 *
Occurrence of hydrocephalus (Yes(reference), No)	0.2696	0.1550-0.4689	<0.001 *

**Table 5:** Summary of Results

Severity of anaemia	
No anaemia: /Hb.95 g/L	624 (71.7%)
Anaemia Hb 80-.95g /L	102 (11.7%)
Severe anaemia: Hb<80g/L	104 (12.0%)
Missing	40.0
Timing of anaemia	
Early anaemia<7 days	183 (21.0%)
Mid-stay anaemia 8-14 days	40.0 ( 4.6%)
Late anaemia>15day of stay	27.0 (3.1%)
Missing	620
Modified Rankin Scale score	
0	181(20.8%)
1-3	405 (46.6%)
4-5	138 (15.9%)
6	142 (16.3%)
Missing	4 (0.4%)
Death in ICU	
No	795 (91.4%)
Yes	75 (8.5%)
Direction of discharge	
Deceased	142 (16.3%)
Neuro-rehabilitation centre/ hospital	213 (24.5%)
Home	497 (57.1%)
Other	18.0 (2.1%)

## Discussion

Our study aimed to investigate whether anaemia in SAH was associated with greater mortality and morbidity. The implications of this study for practice are significant, as anaemia is a measurable and modifiable variable in intensive care settings that can be corrected. Several studies [8,21-23] have estimated the occurrence of anaemia in SAH, ranging from 4% to 47%, although the definitions and terms of reference differ slightly.

Our results, therefore, reinforce previous studies of a similar design, such as Naidech et al. [22], although their study had a smaller sample size and used the Fisher grading scale for SAH instead of WFNS. Naidech et al. concluded that patients with higher initial Hb values had improved outcomes, which complements the results of our study in showing that patients who did not have anaemia tend to have better outcomes in the context of their SAH. English et al. [23] observed that a fifth of patients with SAH received RBCT, unlike our study, where only 64 (7.36%) patients with SAH were transfused. Although English et al. statistically adjusted for confounders and concluded that anaemia was related to poor outcomes of SAH, they could not statistically demonstrate that RBCT improved outcomes [23]. They did demonstrate that it was not correlated with negative outcomes. The SaHaRa trial demonstrated that a liberal transfusion strategy did not result in a lower risk of unfavourable neurological outcomes, and was associated with a better 12-month MRS score and incidence of vasospasm, which helps equipoise of treating the anaemia with transfusions [15].

Ayling et al. demonstrated that anaemia was present at admission in 5% of patients presenting with SAH and in 29% of patients on days one to three of admission [8]. In our study, as several patients were referred from other hospitals, Hb values at admission were unavailable in all cases, and pre-admission anaemia could not be reliably established. Similar to English [23], Ayling et al. also found that RBCT of anaemic patients did not improve long-term outcome or mortality rates, but RBCT of patients with a haemoglobin concentration >100 g/L was associated with improved neurological outcomes [8]. Naidech et al.'s randomised controlled trial of patients with aneurysmal SAH (aSAH) showed that MRS outcomes at 14 and 28 days were better in patients whose Hb was maintained at 115g/L, compared to patients whose Hb was less than 100g/L [22]; this is corroborated by the findings of the current study.

Decreased cerebral oxygen delivery in SAH leads to several associated complications, including disseminated intravascular coagulation (DIC) and vasospasm due to a buildup of nitric oxide synthases in red blood cells, which exacerbates the reduced oxygen delivery and causes further ischaemia and infarction. Furthermore, even in the absence of macrovascular ischaemia, diffusion hypoxia can result from brain injury, necessitating further neuroprotection through optimised oxygen delivery [11]. This premise is also supported by other studies, which demonstrate that a transfusion resulted in a 15% rise in Hb and arterial oxygen content [24]. Likewise, Kurtz et al. demonstrated, using microdialysis sampling and neurological monitoring, that anaemia was associated with increased cerebral tissue hypoxia and distress (adjusted OR 1.7 (95%CI 1.1-2.4); P = 0.01 for every unit decrease) [25]. Our study clinically suggests that patients who had anaemia had a higher risk of worse MRS outcomes (4-6), indicating significant disability and/or death (p<0.001). 

The SaHaRa randomised controlled trial demonstrated that in patients with aSAH and anaemia, a liberal strategy of RBCT did not result in a lower risk of an unfavourable neurological outcome at 12 months compared to a restrictive strategy and has added strength to the argument that a liberal transfusion threshold might not pose harm in aSAH [15]. Our study's findings of anaemia, with a definition of Hb<95, which was lower than that of the SaHaRa trial, being associated with greater morbidity and mortality, support the recommendation for a liberal transfusion threshold. However, more research is required in the context of the United Kingdom and European healthcare systems and patient blood management protocols. There is evidence to suggest that younger patients, those presenting in better clinical condition, and those without CT evidence of large stroke demonstrate the highest capacity for delayed recovery [26]. 

Limitations and future directions

One of the limitations of our study is that the number of units of blood transfused was not captured accurately, which would have helped determine if there was a correlation between MRS and the number of units needed for transfusion. Furthermore, we suggest that there is a need to investigate whether the lowest Hb category is significant if adjusted for the severity of bleed (WFNS). While we excluded patients with haematological malignancies, confounding factors such as non-malignant haematological disorders and the use of anticoagulants for pre-existing medical conditions may have affected results, which were not screened for exclusion, potentially limiting our findings; however, this study's reliability was increased through a large population size, thorough data collection and wide inclusion criteria. Anticoagulants can increase the risk of bleeding events, and non-malignant haematological disorders can play a role in contributing to anaemia in SAH, which must be addressed.

One of the possible limitations of our study is that a substantial minority of poor-grade SAH patients experience delayed recovery beyond the 28-day point we used as our primary outcome parameter. Although a 30-day MRS score is a strong indicator of a 90-day MRS score [27], patients may go on to experience a reduction in functional ability and MRS score at 12 months [28]. There is a clear indication that future studies should utilise a longer follow-up period if resources permit.

We suggest future studies focus on determining whether blood transfusion can improve outcomes through a much larger multi-centre, multinational prospective study after the publication of the SAHaRA trial [15], as there is a risk of it being underpowered [29,30]. We propose that future studies could focus on the comparison of early and late anaemia in SAH, the timing of transfusion in relation to the incidence of anaemia, and how this impacts secondary outcomes. The optimal trigger point for RBCT for these patients also needs to be determined, as guidelines on transfusion thresholds still tend to vary significantly.

## Conclusions

Anaemia is a recognised complication after the critical illness following an SAH, and it is associated with a significant increase in risk of death and severe disability. The results of the study indicate that Hb in patients with SAH should be monitored closely and corrected (if Hb<95g/L), and suggest a liberal transfusion threshold may be beneficial; however, further evidence is required to ensure that blood transfusion is the optimal method to treat anaemia in this population of patients and further study into the causative effect between anaemia and increased mortality and morbidity is required. Looking forward, further study is required to determine if there is an optimal Hb level to trigger RBCT in patients with SAH; as yet, there is not enough evidence to provide clear recommendations and national or international guidance regarding a specific Hb target and transfusion strategy in this patient population.
